# Recycling Polyethylene-Rich Plastic Waste from Landfill Reclamation: Toward an Enhanced Landfill-Mining Approach

**DOI:** 10.3390/polym11020208

**Published:** 2019-01-26

**Authors:** Roberto Avolio, Francesco Spina, Gennaro Gentile, Mariacristina Cocca, Maurizio Avella, Cosimo Carfagna, Gianluigi Tealdo, Maria Emanuela Errico

**Affiliations:** 1 Institute for Composites, Polymers and Biomaterials (IPCB-CNR), via Campi Flegrei 34, 80078 Pozzuoli, Italy; gennaro.gentile@ipcb.cnr.it (G.G.); mariacristina.cocca@ipcb.cnr.it (M.C.); maurizio.avella@ipcb.cnr.it (M.A.); carfagna@unina.it (C.C.); mariaemanuela.errico@ipcb.cnr.it (M.E.E.); 2 Department of Civil, Architectural and Environmental Engineering, University of Naples Federico II, Piazzale V. Tecchio 80, 80125 Napoli, Italy; spinafrancesco91@gmail.com; 3 IREOS S.p.a., Via Stefano Turr 165, 16147 Genova, Italy; g.tealdo@ireosweb.com

**Keywords:** recycling, mixed plastics, polyolefins, landfill mining, polymer composites, ball milling

## Abstract

In the frame of a circular economy, the maximization of secondary raw-material recovery is necessary to increase the economic and environmental sustainability of landfill mining and reclamation activities. In this paper, the polyethylene-rich plastic fraction recovered from the reclamation of an abandoned industrial landfill (landfill-recovered plastic, LRP) has been characterized through spectroscopic, thermal, morphological, and mechanical analyses. Then, an economically viable valorization and recycling strategy was set up. The effectiveness of this strategy in the enhancement of LRP properties has been demonstrated through morphological and mechanical characterizations.

## 1. Introduction

Landfill reclamation describes a series of activities consisting of the excavation of closed landfills and subsequent waste retreatment. The first aim of such activities is the removal of environmental hazards posed by landfills, in particular for sites commissioned before the introduction of safety regulations [1], and the reclamation of land for other uses, with material recovery often limited to metals and methane [2]. The importance of mining activities coupled with reclamation has, however, gained increasing attention in the frame of the circular economy. The concept of Enhanced Landfill Mining (ELM) has recently been proposed as “the safe conditioning, excavation, and integrated valorization of landfilled waste streams as both materials and energy, using innovative transformation technologies and respecting the most stringent social and ecological criteria” [3]. In this view, new landfill-mining operations must follow an integrated approach that maximizes waste recovery (as secondary raw materials or energy) and land reclamation, reducing to a minimum the environmental impact of the process and the amount of relandfilled, unrecoverable wastes. The implementation of ELM is an ambitious societal and environmental challenge, but also a huge opportunity, considering that there are at least 500,000 landfill sites in Europe [3], containing an enormous amount of potential resources. By clever management of raw material and energy recovery, the integrated ELM approach could balance a large part of the (high) cost of excavation and waste retreatment, making the reclamation process self-sustaining.

The composition of materials recovered from landfill excavation can show large variability depending on geographic location, main use (municipal, industrial), and period of activity [4]. In particular, plastic materials are one of the largest fractions in most landfills, amounting up to 25 wt% in some sites [4,5], and a high content of plastics is expected in all landfills commissioned before the introduction of waste-management directives, e.g., packaging waste [6]. Management of waste plastics is one of the key points to be addressed for the sustainability of the mining/reclamation process.

The reprocessing of excavated plastics is particularly challenging due to the high presence of organic and inorganic contaminants, large variability in polymer types and grades, and the possible occurrence of chemical and thermo-oxidative degradation [4]. For these reasons, energy recovery is often indicated as the most suitable route for the valorization of landfill-recovered plastic (LRP) [7]. LRP composition depends on the nature of the landfill (municipal, industrial) and on changes in the waste-stream input over the years [8]. Polyolefin materials have the highest share in the packaging market [9] and, in particular, a high polyethylene (PE) content is expected [10] in both municipal and industrial waste. However, large compositional variability is usually observed and, as an example, relevant amounts of polyethylene terephthalate (PET), polystyrene (PS), and poly(vinyl chloride) (PVC) [11] are often found in municipal landfills. The first step in reclamation is to separate the waste stream from inert materials (sand, gravel) used in landfilling; one or more washing and sieving stations are usually installed in landfill reclamation sites [12]. Such separation systems can be designed to maximize the recovery of plastics and to remove part of the contaminants, meeting the requirements of energy-recovery technologies like pyrolysis and gasification in terms of, as an example, PVC content [7]. Advanced sorting lines inspired to postconsumer plastic-recycling technologies can, in principle, be installed, increasing sorting efficiency; however, expensive equipment and high operation cost could be economically unsustainable. Moreover, contamination by inorganic soil and dust particles is observed in all excavated materials and LRP washing shows limited effectiveness in the removal of such particles [10].

**Figure 1:**
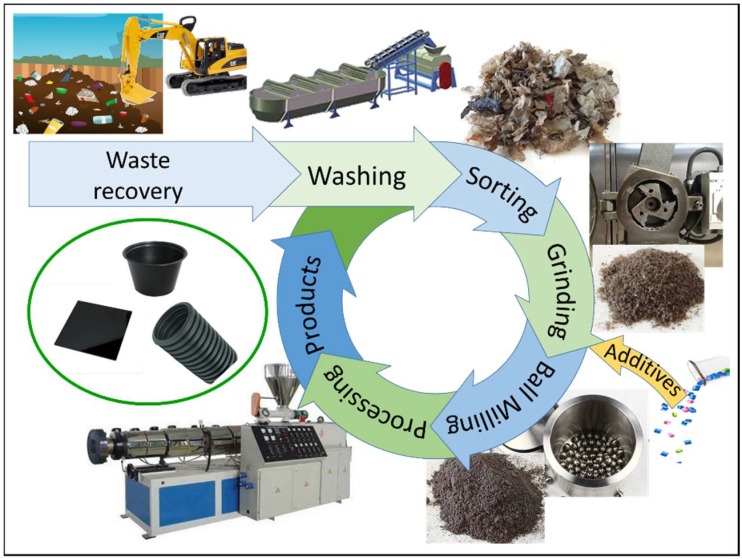
Scheme of the grinding, preprocessing, and processing steps applied to the landfill-recovered plastic (LRP) material.

In this paper, a strategy for valorization through the mechanical recycling of a polyethylene-rich LRP stream recovered from a reclaimed industrial-landfill site has been experimented as schematically illustrated in Figure 1. The process was designed to withstand the relatively high contamination level expected in LRP, and to be robust against compositional variability. Mechanical recycling methods for mixed polyolefin-based plastics have been widely investigated, and a number of effective processing and compatibilization strategies have been developed depending on the composition and presence of fillers [13]. Recently, the ball-milling process (BM) has been proposed as a new, versatile strategy to produce plastic blends and composites with enhanced properties [14]. During the process, the intense mechanical stress exerted onto sample particles can induce not only fine grinding but also an intimate mixing of the different components, leading to better homogenization and dispersion in multiphase materials. Moreover, the strong compressive and shear forces could induce drastic changes in the microstructure of polymers, further influencing the morphology and properties of the resulting materials [15]. Combining the use of solventless BM treatment and a small amount of commercial polymeric additives, the proposed process aims to enhance the dispersion of inclusions and secondary polymeric phases in the matrix while staying flexible and nonspecific, to produce recycled materials with optimized properties.

Before processing, the LRP stream was analyzed by spectroscopic, thermogravimetric, and morphological analyses to evidence its composition and properties. The processing parameters of LRP were then optimized, and the obtained recycled materials were characterized by mechanical, morphological, and thermal analyses.

## 2. Materials and Methods

### 2.1. Materials

A sample of LRP, the plastic-rich, light fraction recovered from landfill reclamation activities, was kindly supplied by IREOS S.p.A. (Genova, Italy). The material was excavated during the reclamation of an abandoned landfill in Portoscuso, Italy, and subjected to a proprietary wet gravimetric selection and washing process. The resulting material was mainly composed of plastic film pieces of various size, clearly stained due to long contact with soil.

Maleated linear low-density polyethylene (MAPE), trade name Compoline CO/LL, with a grafted maleic anhydride content of 1.4 wt% was kindly supplied by Auser Polimeri S.r.l. (Lucca, Italy).

Ethylene-propylene copolymer (EPR), Dutral CO 059, was kindly supplied by Versalis S.p.A. (Milano, Italy).

Maleated ethylene-propylene-diene copolymer (EPDM-g-MA) Fusabond N525, was kindly supplied by DuPont de Nemours Italiana S.r.l. (Milano, Italy).

### 2.2. Processing of LRP-Based Materials

The pristine LRP material was ground in a SM100 rotary knife mill (Retsch GmbH, Haan, Germany), using a bottom sieve with 2 mm openings. Ground LRP was melt-processed in the presence of an amount between 2.5 and 10 wt% of additives (compositions reported in Section 3.2) in a Brabender Plastograph EC batch mixer (Brabender GmbH, Duisburg, Germany) at 190 °C and 60 rpm for 8 min. Materials were then pelletized and compression-molded at 190 °C, 50 bar, obtaining 1.5 and 3 mm thick sheets to be used for subsequent analysis. The ground LRP was processed in the same conditions and analyzed as a reference.

The selected LRP/additive combinations were also subjected to a preprocessing step in a PM100 planetary ball mill (Retsch GmbH, Haan, Germany), using a 500 mL steel grinding bowl and 2 mm steel balls. The ball/sample weight ratio, bowl rotation speed, and grinding time were optimized and set to 10/1, 400 rpm, and 2 h, respectively. Ball-milled powders were processed and molded as described above.

### 2.3. Techniques

Infrared spectra were recorded by means of a Spectrum 100 FTIR spectrometer (PerkinElmer, Waltham, MA, USA), equipped with an attenuated total reflectance accessory (ATR). The scanned wavenumber range was 4000–400 cm^−1^. All spectra were recorded with a resolution of 4 cm^−1^, and 16 scans were averaged for each sample.

Solid-state ^13^C Magic Angle Spinning (MAS) Nuclear Magnetic Resonance (NMR) spectra were collected on a Bruker Avance II 400 spectrometer (Bruker Biospin, Billerica, MA, USA) operating at a static field of 9.4 T, equipped with a 4 mm MAS probe. Ground LRP samples were packed into 4 mm zirconia rotors sealed with Kel-F caps and spun at 10 kHz. Cross-polarization (CP) spectra were recorded with a relaxation delay of 5 s and a contact time of 2 ms under high-power proton decoupling. Spectra were referenced to external adamantane (CH_2_ signal at 38.48 ppm downfield of tetramethylsilane (TMS), set at 0.0 ppm).

Thermogravimetric analysis (TGA) was carried out on a Pyris 1 TGA analyzer (PerkinElmer, Waltham, MA, USA) using air as purge gas and a linear heating ramp from 40 to 800 °C at 10 °C/min. 

Differential scanning calorimetric analysis (DSC) was performed on a TA-Q2000 system equipped with an RCS-90 cooling unit (TA Instruments, New Castle, DE, USA). The instrument was calibrated in temperature and energy with pure indium. About 5 mg of each sample was sealed into aluminum pans and subjected to the following temperature program: heating from 25 to 290 °C; cooling from 290 to −50 °C; and heating from −50 to 290 °C. The heating/cooling rate for all runs was fixed at 10 °C/min.

Tensile tests were performed on dumb-bell specimens (6 mm^2^ cross section, 1.5 mm thickness, 26 mm gauge length) at a cross-head speed of 10 mm/min by using an Instron 5564 testing machine (ITW Inc. Glenview, IL, USA). Young's modulus (E), peak stress (σ_max_), and elongation at break (ε_R_) were calculated as average values over at least 6 tested samples.

Impact tests were performed on an instrumented Ceast Charpy pendulum Resil Impactor (ITW Inc. Glenview, IL, USA), equipped with a DAS 4000 Acquisition System, using an impact energy of 6.7 J and an impact speed of 1.92 m/s. Samples (10.0 mm wide, 3 mm thick, and 60 mm long), with a notch depth to width ratio of 0.3 and a span length of 48.0 mm, were tested at room temperature. For each material, 6 specimens were tested and the average values of resilience and peak force were calculated.

Scanning electron microscopy (SEM) was carried out on a Quanta 200 FEG microscope (FEI, Hillsboro, OR, USA) working in high vacuum mode with an acceleration voltage ranging from 10 to 30 kV and using a secondary electron detector. Before SEM observations, cryofractured surfaces were sputter-coated with an Au/Pd alloy by means of an Emitech K575X sputtering device.

Energy dispersive X-ray analysis (EDX) was carried out in the same SEM by means of an Inca Energy System 250 and an Inca-X-act LN2-free analytical silicon drift detector (Oxford Instruments NanoAnalysis & Asylum Research, High Wycombe, UK), in high vacuum mode at 30 kV acceleration voltage. 

## 3. Results and Discussion

### 3.1. Preliminary Analysis of LRP Composition and Properties

Before processing, several LRP films were randomly selected and analyzed by means of infrared spectroscopy (FTIR).

FTIR analysis was carried out according to the procedure described in Section 2.3 and selected FTIR spectra are reported in the Supporting Information (Figure S1). FTIR analysis qualitatively indicated the large presence of PE fragments and, in a lower amount, polypropylene (PP). In the PE fragments, the amount of carbonyl groups, indicative of thermo-oxidative degradation of the polymer chain, was negligible. Moreover, LRP results were contaminated by inorganic materials, compatible with silicates, probably due to the presence of residual soil not removed by the washing process [10].

Further insight on composition was obtained by solid-state NMR spectroscopy and by morphological and EDX analyses carried out on processed LRP samples.

**Figure 2:**
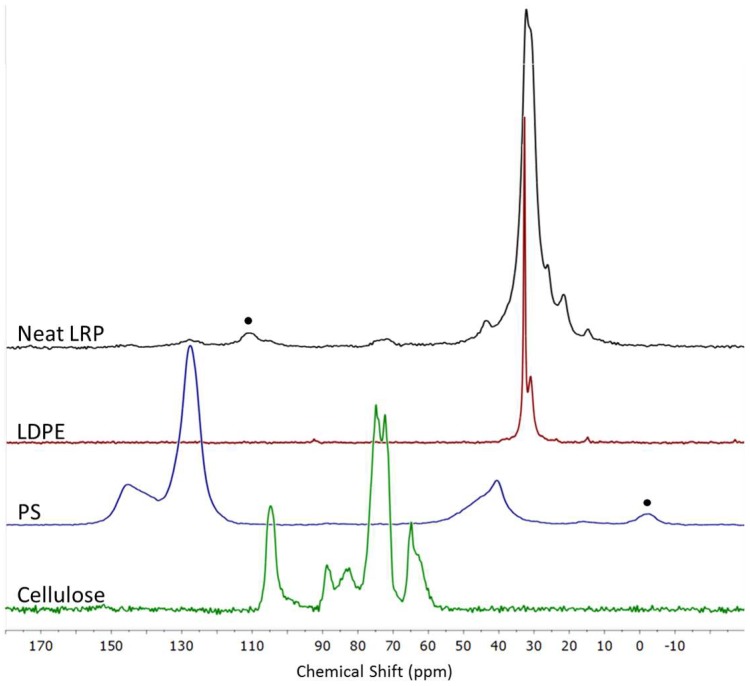
Solid state ^13^C NMR spectrum of LRP, compared with the spectra of low-density polyethylene (LDPE, red), polystyrene (blue), and cellulose (green). Spinning sidebands are marked by a dot.

The ^13^C spectrum of LRP, Figure 2, is dominated by a broad and complex signal centered at about 32 ppm, attributed to PE backbone carbons. This peak is broader than in commercial low-density polyethylene (LDPE), whose NMR spectrum is shown for comparison, probably due to the presence of different PE grades and of some contaminants characterized by aliphatic chains. The peak at 15 ppm is also attributed to PE, namely, to chain branches in LDPE or linear LDPE (LLDPE) [16]. By comparing the relative intensity of branching vs. the main chain peaks in pure LDPE and LRP, it can be assessed that most of the PE contained in LRP is branched. The resonances at 22, 26, and 44 ppm that partly overlapped with the main PE signal are ascribed to PP resonances [17], in agreement with the results of FTIR analysis. At lower fields in the spectrum, signals attributed to cellulosic materials (60–90 ppm and 105 ppm) and to the aromatic ring of polystyrene (130 and 145 ppm) were observed. The main carbon peaks of PVC, expected in the 40–60 ppm range, were not detected. Moreover, no signal was evidenced in the carbonyl region, ruling out the presence of a significant amount of polyester (e.g., PET) and confirming the very low extent of thermo-oxidative degradation in PE. The low resolution and low intensity of the contaminant signals prevented quantitative analysis, but qualitative estimation was performed defining the limit content of the main organic contaminants: PP < 5 wt%; cellulose < 4 wt%; PS < 2 wt%.

Morphological analysis, reported in Supplementary Material, Figure S2, and EDX analysis, Figure S3, confirmed the presence of large lignocellulosic fragments (wood, plant stems), minerals, and some metal particles embedded into the polymeric matrix, and allowed to identify some mineral inclusion as sodium chloride crystals and calcium/aluminum silicates. The amount of inorganic materials in LRP was estimated by analysis of the weight-loss curve recorded by TGA (see also Section 3.4). The residual weight at 700 °C, after the complete degradation of the polymeric matrix [18], was equal to 11% and attributed to inorganic inclusions.

Summarizing, the examined LRP sample was essentially constituted by LDPE, with moderate amounts of PP, cellulosic materials, polystyrene, and soil minerals. A preliminary tensile testing of neat, molded LRP revealed (Table 1) a low elastic modulus, compatible with the values usually shown by LDPE. The low modulus, however, pairs with low strength and very low ultimate elongation, around 10%, in striking contrast with the response of LDPE, which is highly deformable under tension. This behavior can be ascribed to the relatively high amount of rigid contaminants (inorganics, cellulose) detected in LRP that hinder the deformation of the polymeric matrix and induce a fragile behavior.

### 3.2. Additivation, Processing and Mechanical Testing

To improve LRP properties and extend its possible applications, three commercial polymeric additives, selected on the basis of LRP composition, were tested. The aim was to set up a valorization strategy able to enhance and optimize the properties of LRP-based materials, combining simple additives and the use of a planetary ball mill as an advanced preprocessing step. The tested additives are listed below:

- Polyethylene modified with maleic anhydride
  Maleated polyethylene (MAPE), which consists of a PE backbone grafted with polar maleic anhydride groups, whose positive effect on adhesion with inorganic and, in general, hydrophilic fillers is widely recognized [19]. MAPE is employed in PE-based blends and composites to improve the dispersion of different additives and fillers (inorganics, flame retardants, natural fibers) and to increase the interfacial adhesion in such systems [20]. Due to the presence of inorganic and cellulosic contaminants in LRP, MAPE is expected to have a positive impact on mechanical strength and flexibility.
- Ethylene–propylene copolymer
  EPRs, besides their applications in the rubber industry, are well-known impact modifiers for polymers. They have fairly good miscibility with many polyolefins and have also been employed as interfacial agents in PE/PP blends [21]. Their use is then expected to improve the miscibility of different PE fractions among themselves and, with PP, present in a minor amount, increase the flexibility and resilience of LRP.
- Ethylene-propylene-diene modified with maleic anhydride
  Maleated EPDM is mainly used as an impact modifier for engineering plastic formulations, and there are relatively few examples of additive applications for PE-based compounds. In LRP-based materials, EPDM-g-MA could combine the benefits of both adhesion modifier (MAPE) and impact modifier/compatibilizer (EPR) in one product.

A planetary ball mill (BM) preprocessing step was applied to the LRP/additive mixtures showing the most promising properties. The intense mixing promoted by BM treatment is highly suited for the homogenization of heterogeneous mixtures, and can produce synergistic effects in combination with the additives. The implemented BM treatment was carried out at room temperature and, in the frame of a sustainable approach, no solvents or reagents were added. The mild temperature increase induced by friction during the process, typically below 100 °C, was not expected to induce degradation phenomena in the treated polyolefin-based materials. It is worth to note that the used additives and processing methods were selected to enhance the adhesion of the observed inclusion (lignocellulosic, inorganics) with the PE-rich matrix and to improve their dispersion by fine grinding and homogenization. This approach can be adapted to deal with other common contaminants, in particular PET and PVC, which are often encountered in waste-sourced plastics. Maleic anhydride-modified polymers are, in fact, effective adhesion promoters for a broad set of polar surfaces, including PET [22], and similar compounds can improve PVC dispersion in PE [23]. Ball milling conditions can be tuned to optimize size reduction and phase mixing, and BM has already been applied to PVC with interesting effects on morphology [15].

After melt processing, all materials were compression-molded and tested: compositions and codes of all prepared materials are reported in Table 1.

As discussed, neat LRP has a low modulus and low ultimate elongation. The addition of rubbery additives, EPR and EPDM-g-MA, induced a small increase in elongation at break, in particular at 5% of EPR. This result was, however, reached at the expense of the elastic modulus, which decreased by up to 50%, and strength. While lower stiffness could be expected, due to the inherently low modulus of EPR/EPDM, the minor increase in elongation probably indicates that ethylene copolymers did not have strong effects on polymer/polymer (e.g., PE/PP) or on polymer/filler (for the case of EPDM-g-MA) compatibility. This hypothesis was further confirmed by impact-test results that revealed a strong increase of impact toughness in presence of the rubbers. For EPDM-g-MA, toughness was not related to additive content. Apparently, copolymers then showed limited miscibility with the LRP matrix and acted as impact modifiers, segregating in highly deformable rubbery clusters [24] instead of continuously mixing with the matrix.

The addition of MAPE, on the other hand, produced materials with more balanced properties, as a significant increase in elongation was accompanied by an increase in tensile strength and a nondramatic decrease of stiffness. Miscibility of the used MAPE with LRP was expected to be fairly good, as most of the LRP is LDPE. The ability of maleic anhydride groups to improve matrix adhesion with relatively abundant polar inclusions, minerals, and cellulose, produced stronger interfaces, able to withstand higher deformation than observed in neat LRP [20]. This finding is in agreement with the results of morphological analysis, illustrated in next section. MAPE also increased impact toughness, in particular, over 5% of addition, probably due to the same adhesion-enhancement effect. Loose interfaces can, in fact, represent a preferential path for fracture propagation.

MAPE-containing materials, showing better properties, were selected to experiment BM pretreatment, with neat LRP as a control. A strong effect of BM on properties was evidenced on a BM LRP sample, as pretreatment was able to increase elongation at break twofold and peak stress by 20%. Even stronger was the effect of BM on MAPE-containing formulations that attained an elongation of 47%, preserving the same strength of the reference BM LRP sample and a slightly lower elastic modulus. These findings are essentially attributed to the size reduction and fine dispersion of inclusions, particularly of cellulosic particles, promoted by BM, as discussed in next section [25,26]. The small particle size, coupled with good interfacial adhesion promoted by MAPE, do not hinder the elongation and flow of the polymeric LRP matrix under tension, enabling a relatively high deformation before failure. Similar behavior was previously observed in cellulose/polymer composites [27,28], showing that elongation is increased by balancing the size and amount of the filler, and the strength of interfacial adhesion.

Interestingly, the impact strength of the BM LRP sample showed the opposite behavior with respect to ultimate elongation, as it was greatly reduced by the ball-milling treatment. This was not observed in the MAPE-containing materials, whose impact resilience was only marginally affected by the pretreatment. Again, this difference can be related to the different strength of interfaces in neat BM LRP and MAPE-containing samples. The fine dispersion of inclusions lacking adhesion with the matrix produces a higher content of weak interfaces that ease fracture propagation, and reduces the deflection effect that large inclusions may provide. This fact has a lower impact on properties in the presence of MAPE due to the better interfacial adhesion that controls the mechanical properties.

### 3.3. Morphological Analysis

Mechanical-testing findings point out the important influence of morphology on properties in complex multiphase materials. Analysis of sample morphology by SEM highlighted the different effects of additives and BM treatment, shedding light on the origin of the recorded mechanical response.

**Figure 3:**
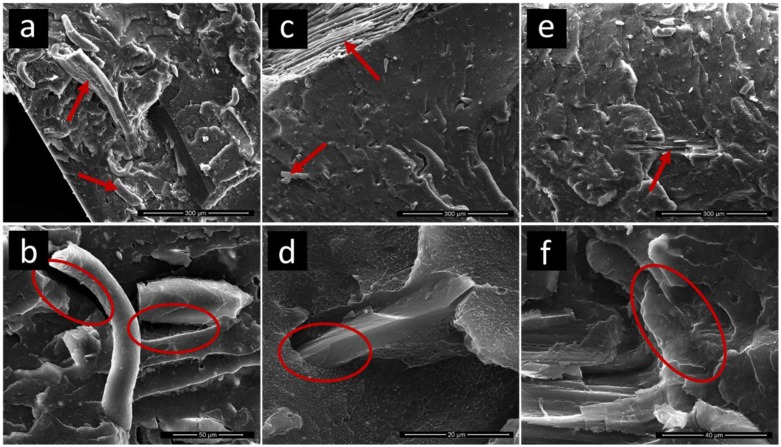
Micrographs of cryofractured surfaces of (**a**,**b**) 2.5 ethylene-propylene copolymer (EPR), (**c**,**d**) 2.5 maleated ethylene-propylene-diene copolymer (EPDM-g-MA), and (**e**,**f**) 5 maleated linear low-density polyethylene (MAPE). Main inclusions are indicated by arrows, while relevant polymer/particle interface areas are highlighted by ellipses.

Micrographs of representative additivated samples are reported in Figure 3.

Lignocellulosic inclusions with broad size distribution (tens to hundreds of µm) were observed in all samples together with µm-sized fibers and particles; the largest inclusions are indicated by red arrows in Figure 3a,c,e. Adhesion of the polymer matrix to the polar inclusions in the 2.5 EPR sample (Figure 3a,b) was poor, as evidenced by a close inspection of the interfacial regions. The mechanical load, applied during the fracture, in fact induced extensive failure of the matrix/filler interface (debonding). Somewhat better adhesion was observed in the material containing EPDM-g-MA (Figure 3c,d), but the adverse effect on the mechanical response, as discussed in the previous section, suggests that the interfacial layer is not effective in transferring stress during tensile testing. This finding further confirms the hypothesis of nonoptimal miscibility of the additive with the matrix and points out its preferential localization at the surface of polar inclusions [24,29].

In the presence of MAPE, a strong improvement of interfacial adhesion was obtained, as demonstrated by the continuous interface observed in Figure 3e,f. In particular, Figure 3e shows a wooden inclusion (indicated by a red arrow) that was apparently broken apart during fracture instead of being pulled away, indicating effective stress transfer across the interface and supporting the higher tensile strength shown by MAPE-containing materials.

**Figure 4:**
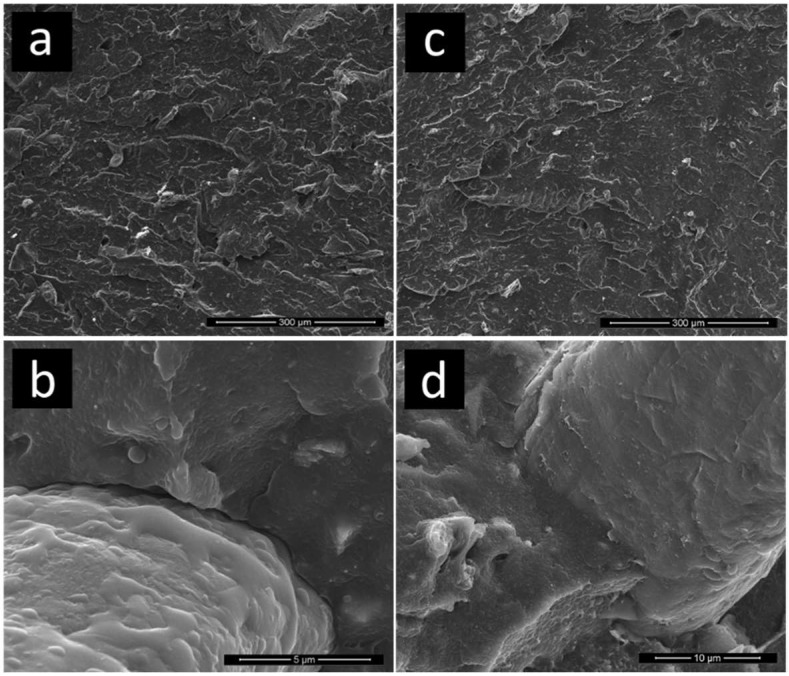
Micrographs of cryofractured surfaces of (**a**,**b**) neat LRP and (**c**,**d**) ball-mill (BM) 5 MAPE.

The morphology of BM-treated samples is reported in Figure 4.

The main morphological difference between untreated and BM-treated samples is the much finer size of the inclusions. Comparing the micrographs of untreated and BM-treated samples (Figure 2 and Figure 4, respectively), the complete absence of lignocellulosic and inorganic particles larger than ~100 µm can be evidenced as a consequence of BM.

It is to be noted that similar size reduction if pursued in a rotary or knife mill would most probably overheat the polymeric matrix, leading to degradation and/or melting. It can be observed that MAPE also effectively enhanced the adhesion of the LRP matrix to polar particles/fibers in BM-treated materials, as demonstrated by the almost-continuous interface observed in Figure 4c,d. These findings allow to rationalize the results of tensile/impact tests, and to point out the importance of the preprocessing step combined with additives in the valorization of waste-sourced plastics.

### 3.4. Thermal Analyses

**Figure 5:**
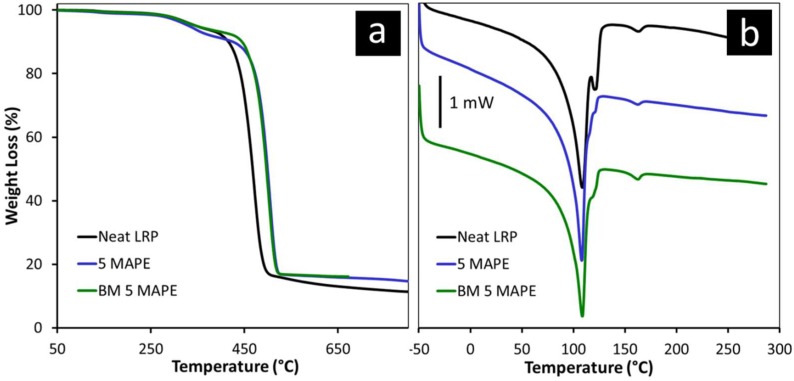
(**a**) Thermogravimetric analysis (TGA) weight-loss curves and (**b**) differential scanning calorimetric analysis (DSC) traces of selected LRP-based materials.

Thermogravimetric and calorimetric analyses were carried out on LRP- and MAPE-containing samples, showing the best balance of properties, to investigate the effects of additive and treatment on thermal properties. DSC and TGA traces are illustrated in Figure 5.

TGA weight-loss curves confirm the presence of high residue that can essentially be attributed to the presence of inorganic particles. The first weight-loss step, recorded in the 200–400 °C range, was attributed to the thermal degradation of cellulosic material, while the following step is related to the degradation of the polymeric matrix [30]. Interestingly, the main weight-loss process was shifted to about 30 °C toward higher temperatures in MAPE-containing samples. This strong effect can be related to the synergy between inorganic particles that are able to increase the thermal stability of polymers, and improved compatibility with the matrix promoted by the additive. In fact, improvement in thermal stability is often observed in polymers filled with silicaceous particles, similar to soil-derived particles contained in LRP materials [31,32]. The extent of stabilization is related to their dispersion and to the interactions established with the organic matrix [32].

Differential scanning calorimetry confirmed the presence of different PE grades inside LRP, as revealed by the multiple fusion peak shown in Figure 5b, with a probable predominance of LDPE that is characterized by a lower melting point [33]. The melting peak of PP can also be observed at ~160 °C. A reduced intensity of the melting peak at 120 °C, attributed to the melting of high-density polyethylene (HDPE), was observed in MAPE-containing samples. This finding can be related to the high miscibility of MAPE with the linear HDPE fraction, hindering to some extent the crystallization of HDPE as a separate phase [34,35].

## 4. Conclusions

A plastic rich fraction (LRP), recovered from the reclamation of an abandoned industrial landfill, was characterized and processed. LRP composition was studied by means of infrared, NMR spectroscopy, and thermogravimetric analysis. The main component of LRP was identified as branched PE, either LDPE or LLDPE; a relatively high presence of organic (PP, cellulose) and inorganic (soil minerals, salts, metal particles) contaminants was also revealed. The effects of such contaminants on the mechanical properties of LRP were pointed out, and a valorization strategy was set up. In particular, LRP-based recyclates were produced by the combined use of a solventless ball-milling treatment and properly selected polymeric additives. The effect of the proposed strategy on the LRP properties was assessed and rationalized by means of tensile, impact, morphological, and thermal analyses. The addition of MAPE, combined with BM treatment, led to a fourfold increase in ultimate elongation, and a 20% increase in tensile strength, with respect to neat LRP. 

The obtained results demonstrate that the proposed process represents an effective valorization strategy for landfill-sourced plastics. This approach makes use of simple additives and of a flexible BM treatment, and can be adapted to deal with other contaminants and secondary polymeric phases, opening the way for further implementation of the Enhanced Landfill Mining concept.

## Figures and Tables

**Table 1:** Composition and code of all prepared materials, results of mechanical testing: elastic modulus (E), tensile strength (σ_max_), elongation at break (ε_R_), impact resilience (R) *.

Code	Additive	E (MPa)	σ_max_ (MPa)	ε_R_ (%)	R (kJ/m^2^)
Neat LRP	-	280 ± 15	8.6 ± 0.4	11 ± 2	7.4 ± 0.5
2.5 EPR	EPR 2.5%	188 ± 7	8.1 ± 0.2	19 ± 5	9.5 ± 0.7
5 EPR	EPR 5%	150 ± 10	7.4 ± 0.2	24 ± 5	15 ± 4
2.5 EPDM	EPDM-g-MA 2.5%	151 ± 7	7.6 ± 0.4	17 ± 4	15 ± 2
5 EPDM	EPDM-g-MA 5%	130 ± 14	6.6 ± 0.9	14 ± 2	18 ± 1
2.5 MAPE	MAPE 2.5%	259 ± 9	10.4 ± 0.5	19 ± 4	8.9 ± 0.7
5 MAPE	MAPE 5%	188 ± 4	9.9 ± 0.4	23 ± 9	11.1 ± 0.9
10 MAPE	MAPE 10%	198 ± 7	9.7 ± 0.3	25 ± 9	14.7 ± 0.9
BM LRP	-	273 ± 8	10.4 ± 0.1	26 ± 6	4.9 ± 0.4
BM 2.5 MAPE	MAPE 2.5%	202 ± 7	9.8 ± 0.1	47 ± 4	9.5 ± 0.3
BM 5 MAPE	MAPE 5%	221 ± 9	10.3 ± 0.4	45 ± 5	11.2 ± 0.8

* LRP: landfill-recovered plastic; EPR: ethylene-propylene copolymer; EPDM: ethylene-propylene-diene monomer; MAPE: Maleated linear low-density polyethylene; BM: ball-milling process; MA: maleic anhydride; EPDM-g-MA: Maleated ethylene-propylene-diene copolymer.

## Supplementary Materials

The following are available online at http://www.mdpi.com/2073-4360/11/2/208/s1. Figure S1: Example of the ATR FTIR spectra recorded on film fragments selected before grinding and processing; Figure S2: SEM micrograph of cryofractured LRP; Figure S3: Elemental composition from EDX analysis.
