# Supplementary material for: Recycling Polyethylene-Rich Plastic Waste from Landfill Reclamation: Toward an Enhanced Landfill-Mining Approach
Source: Polymers (Basel). 2019 Jan 26;11(2):208. doi: 10.3390/polym11020208 (PMC6419269; doi:10.3390/polym11020208)
Supplement: Supplementary file 1 [file polymers-11-00208-s001.pdf]

Supplementary Materials: Recycling of Polyethylene-Rich Plastic Wastes from Landfill Reclamation: Towards an Enhanced Landfill Mining Approach

Roberto Avolio *, Francesco Spina, Gennaro Gentile, Mariacristina Cocca, Maurizio Avella, Cosimo Carfagna, Gianluigi Tealdo and Maria Emanuela Errico

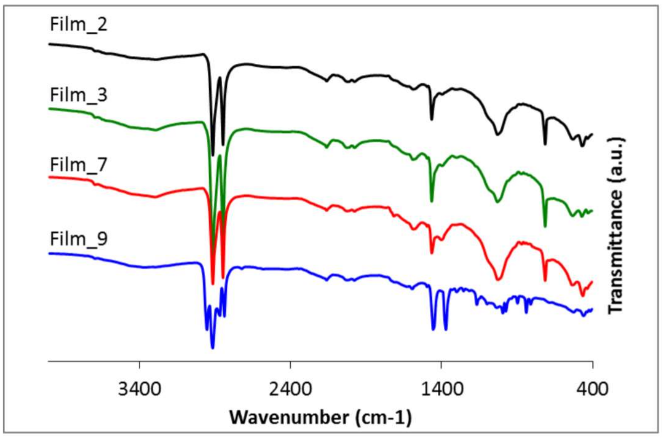

Figure S1. Example of the ATR FTIR spectra recorded on film fragments selected before grinding and processing. While most of the films tested are made of polyethylene (samples 2, 3 and 7), also polypropylene was encountered (sample 9). The broad peak centred at 1000 cm^{-1}, observed in most samples, was attributed to siliceous materials from soil contamination.

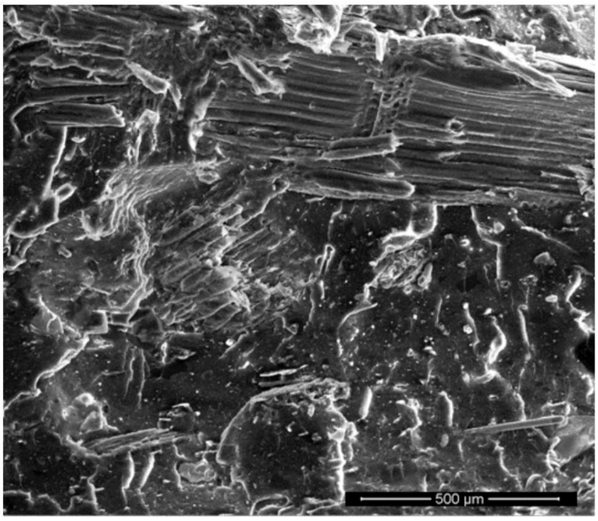

Figure S2. SEM micrograph of cryo-fractured LRP, evidencing a large wooden inclusion and high contrast mineral particles embedded into the matrix.

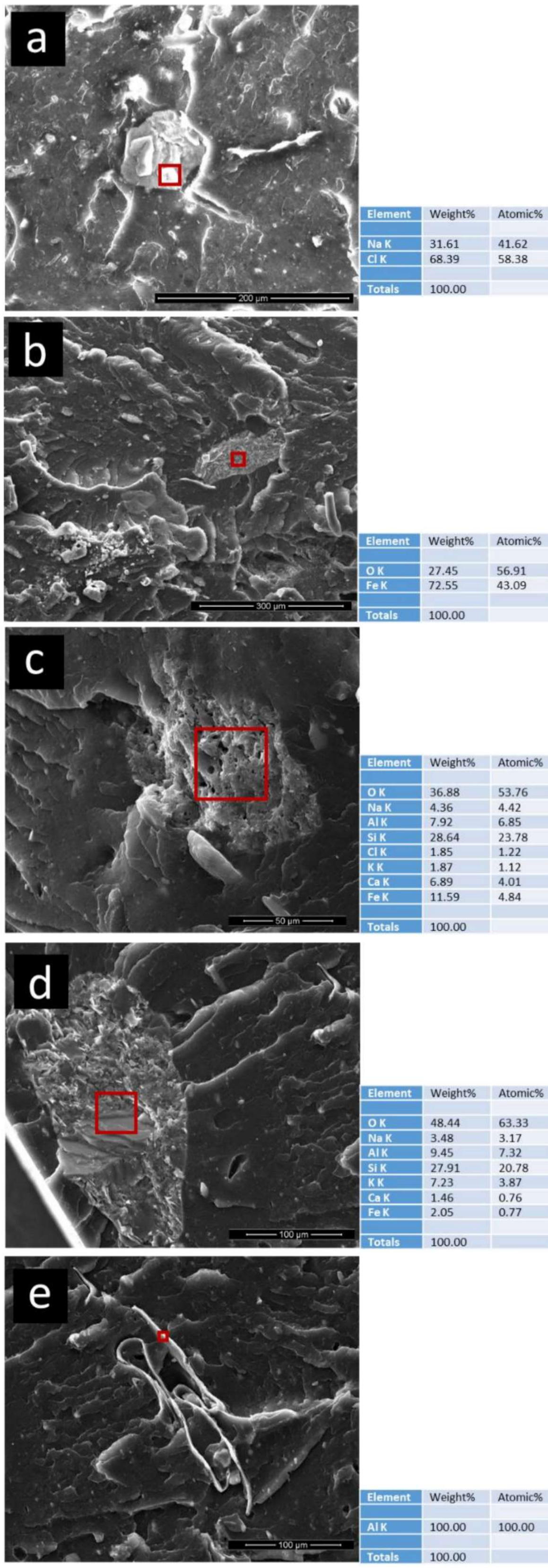

(a)

Element	Weight%	Atomic%
Na K	31.61	41.62
Cl K	68.39	58.38
Totals	100.00	

(b)

Element	Weight%	Atomic%
O K	27.45	56.91
Fe K	72.55	43.09
Totals	100.00	

(c)

Element	Weight%	Atomic%
O K	36.88	53.76
Na K	4.36	4.42
Al K	7.92	6.85
Si K	28.64	23.78
Cl K	1.85	1.22
K K	1.87	1.12
Ca K	6.89	4.01
Fe K	11.59	4.84
Totals	100.00	

(d)

Element	Weight%	Atomic%
O K	48.44	63.33
Na K	3.48	3.17
Al K	9.45	7.32
Si K	27.91	20.78
K K	7.23	3.87
Ca K	1.46	0.76
Fe K	2.05	0.77
Totals	100.00	

(e)

Element	Weight%	Atomic%
Al K	100.00	100.00
Totals	100.00	

Figure S3. Elemental composition from EDX analysis of some inclusion observed in LRP materials. Only the most relevant elements have been included in the calculation. It was possible to identify sodium chloride (**a**), oxidated iron (**b**), rocks (**c**,**d**) and aluminum foil (**e**).
